# The Role of Metal Nanoparticles in Promoting Photocatalysis by TiO_2_

**DOI:** 10.1007/s41061-022-00373-x

**Published:** 2022-03-03

**Authors:** Michael Bowker, Christopher O’Rourke, Andrew Mills

**Affiliations:** 1 School of Chemistry, Cardiff Catalysis Institute, Cardiff University, Cardiff, CF10 3AT UK; 2 Catalyst Hub, RCAH, Rutherford Appleton Lab, Harwell, UK; 3 Max Planck–Cardiff Centre on the Fundamentals of Heterogeneous Catalysis FUNCAT, School of Chemistry, Cardiff Catalysis Institute, Cardiff University, Main Building, Park Place, Cardiff, CF10 3AT UK; 4 School of Chemistry and Chemical Engineering, Queen’s University Belfast, Belfast, BT9 5AG UK

**Keywords:** Photocatalysis, Metal nanoparticles, Solar energy, Photoreforming, Photooxidation, Hydrogen production

## Abstract

In this review, we highlight the role played by metal nanoparticles (NPs) in photocatalytic oxidation with titania as a support. This is presented in two parts, namely, partial photo-oxidation in which an organic sacrificial agent is oxidised in anaerobic conditions to produce hydrogen (photo-reforming), and photo-oxidative mineralisation of organics in aerobic conditions. We present some rules for such reactions that dictate which organic molecules can react readily, and which metals are likely to be useful for such reactions. Generally, the presence of metal NPs enhances enormously the ability of titania to yield hydrogen from photo-reforming, and a wide range of molecules can be used, including biomass. The metal NPs most used are those that are easily reduced, that is, the precious metals. The large enhancement in rate seen with metal for hydrogen production is not so extreme for the oxidation reactions, but is still significant. An important factor in all of this catalysis is the nature of the interaction between the metal NPs, which can play a multiplicity of chemical and electronic roles, and the photoactive support. A sharp dependency of rate on loading of metal is found, with maximum rates at ~0.5–2 wt% loading, depending on the metal used. The source of this dependency is the bifunctional nature of the system, in which the intimacy of both materials is crucial to performance. This rate variation is linked to the interface between the two, which is then linked to the size of the metal NPs. In fact, the rate is proportional to an area adjacent to the metal particles that we call the expanding photocatalytic area and overlap (EPAO) kinetic model. This model describes the dependence well. Rising rates with increasing coverage of particles is associated with increase in this total area but, at the maximum, these areas overlap and at higher loadings the available active area diminishes, reproducing the observed behaviour well.

## Introduction

**Fig. 1:**
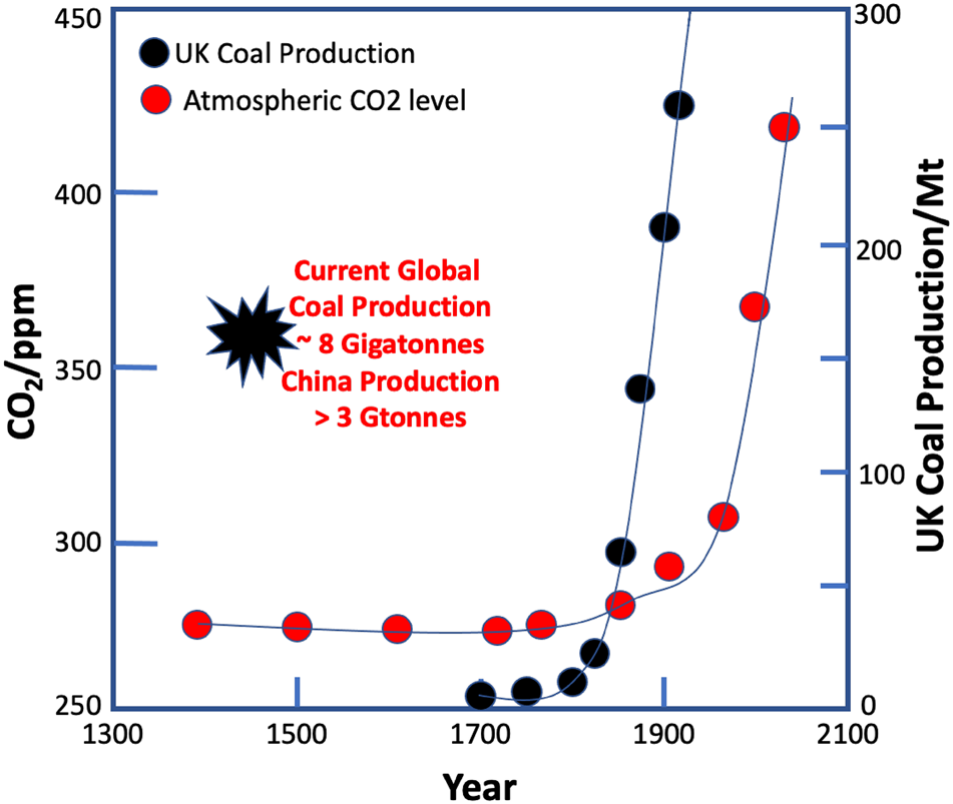
Showing that the rise in atmospheric CO_2_ levels is closely linked to the beginning of industrialisation and the use of coal-fired steam engines associated with it [1, 2]

The global climate is threatened by the activities of mankind. Global warming resulting from the excessive use of fossil fuels and consequent increase of CO_2_ in the atmosphere has now increased by about 60% compared with pre-industrial levels. Figure 1 shows the relationship between CO_2_ level in the atmosphere [1] and coal production at the beginning of the industrial revolution [2] and shows the close relationship between coal burning and global warming. The acceleration of CO_2_ levels in more recent times, from around 310 ppm in 1960 to about 420 ppm now, is still associated with industrialisation around the world, linked mainly with economic growth in the far East [3]. In Western countries, especially the European Union (EU), coal production has declined significantly in very recent times, and the United Kingdom (UK) has essentially none. And yet mankind persists in digging up dirty coal and burning it. The negative effect of this warming has been seen just recently, in the last year, with record temperatures in Canada and deadly floods in Germany and China. We can have no doubt that Nature will correct the negative impact of humans on the atmosphere in the Le Chatelier sense, that is, when a constraint is applied to a system (at equilibrium), the system will respond to re-balance it. The constraint in this case might be seen as the human race.

So, new technologies are required to change the energy profile of humans. We need to develop, and have already developed, some of these—for instance solar power and wind power—and their application is expanding. However, these sources are intermittent and a variety of routes for storing their power at peak production times, for use at low production times, is being considered and developed. One of these is hydrogen production by electrolysis, with subsequent storage in a denser chemical form, such as methanol (Eq. 1) or ammonia. These may then be directly used as fuel, or the hydrogen can be re-extracted from them.

1 $${\text{CO}}_{{2}} + {\text{ 3H}}_{{2}} \to {\text{CH}}_{{3}} {\text{OH }} + {\text{ H}}_{{2}} {\text{O}}.$$

In this article, we consider another way to produce hydrogen, by direct photocatalysis. This can be done using water only, though much of the work in the literature makes use of sacrificial agents to give water splitting, and at least some the hydrogen can also often be produced from the sacrificial agent. For example, and as described in more detail below, methanol can be photo-reformed with water to produce hydrogen from both molecules. This then is also a route back from methanol synthesis from CO_2_ and H_2_ in which 33% of the hydrogen is lost as water, and which then regains that hydrogen back. This may be useful in relation to storage/de-storage systems. Thermally, the reaction is a little difficult since it is endergonic, but is enabled using sunlight as energy input, as can be seen below.

Besides the use of photocatalysis in the energy and chemicals scene, it can also be used to remove pollutants. Despite the well-established nature of the subject area and the development and use of a wide variety of different materials, semiconductor photocatalysis (SPC), continues to be dominated by the photocatalyst, titanium dioxide, TiO_2_, as it is physically, chemically and photochemically stable, photocatalytically active and inexpensive [4]. Indeed, TiO_2_ is the photocatalyst used in all current commercial photocatalytic products, which include self-cleaning glass [5], concrete [6], plastic tent/awning/curtain materials [7, 8], tiles [9] and paint [10–12]. Thus, this review will focus on the role of metal nanoparticles (NPs) in promoting photocatalysis by TiO_2_, usually P25 TiO_2_—which is a 80:20 mixture of anatase and rutile TiO_2_, with a specific surface area of ca. 50 m^2^ g^–1^ [13]—unless stated otherwise.

## Hydrogen Production

### Introduction

The modern era of photocatalysis for hydrogen production perhaps began with the much-cited, but extremely brief, note of Fujishima and Honda [14, 15]. This proposed oxygen evolution from a Pt and reduced TiO_2_ hybrid electrode system with electrolyte present, though with no data for gas evolution: this was photo-electro-catalysis. It is notable that Sato and White [16, 17] reported that hydrogen evolution could be observed when light is admitted to a reduced TiO_2_ sample, but this was due simply to a photo-induced re-oxidation of the titania. However, they did show evidence of water photolysis when metal, in their case Pt, was present on the TiO_2_ [17]. This theme then continued over the years, with metals, often Pt, used as so-called co-catalysts, and reviews of this type of work are given elsewhere [18–20].

Hence the following sections report on hydrogen production using such metal-doped catalysts.

### Hydrogen Production Using Hole Scavengers and Metal/TiO_2_ Catalysts

**Fig. 2:**
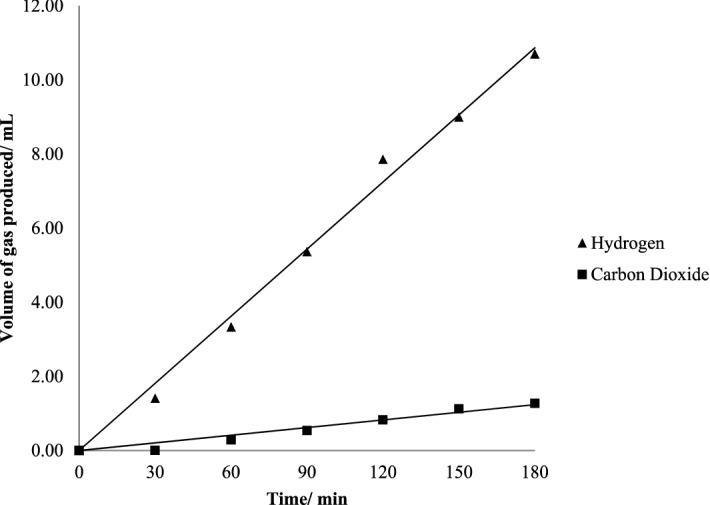
H_2_ and CO_2_ evolution from methanol/water reforming. Using a 0.5 wt% Pd loading catalyst in the liquid phase with 100 ml water and 1% vol methanol. Reprinted with permission from [28]; Copyright 2021 IOPscience

**Fig. 3:**
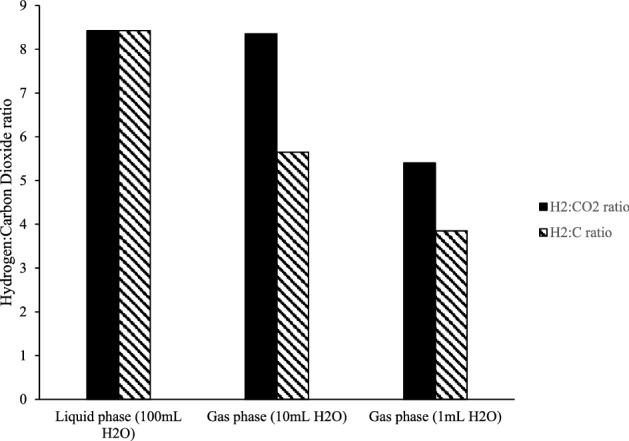
The ratio of H_2_:CO_2_ evolution, and H_2_: (CO_2_ + CO) after 3 h photocatalysis, using different volumes of water. As the water volume decreases, so the reservoir for CO_2_ absorption into the liquid phase diminishes, and so the ratio of gas evolution approaches that of stoichiometry (Eq. 2). Reprinted with permission from [28]; Copyright 2021 IOPscience

Most of the work in this area uses methanol as the hole scavenger, combined with a photoactive support, such as titania, which then limits the useable wavelength of solar radiation, and a so-called co-catalyst, usually metal NPs [21–27]. An example of results from such a system is given below, in this case with Pd as the metal (Fig. 2) [28]. Here it can be seen that both hydrogen and CO_2_ are evolved coincidently. The catalyst is stirred in the liquid phase with water and methanol, with an inert atmosphere above. Note, though, that if the ratio of the gas phase volume above the liquid to the liquid volume is high, then most of the CO_2_ will dissolve and will be observed to be under-stoichiometric in the gas phase [28, 29], as can be seen in this figure. However, if the solution volume is reduced, then the reservoir for CO_2_ absorption is reduced and so more CO_2_ is seen (Fig. 3).

The stoichiometry of the photocatalytic reaction is as follows:

2 $${\text{CH}}_{{3}} {\text{OH }} + {\text{ H}}_{{2}} {\text{O}} \to {\text{CO}}_{{2}} + {\text{ 3H}}_{{2}} .$$

Here, two moles of hydrogen derive from methanol and one from, effectively, water splitting.

**Fig. 4:**
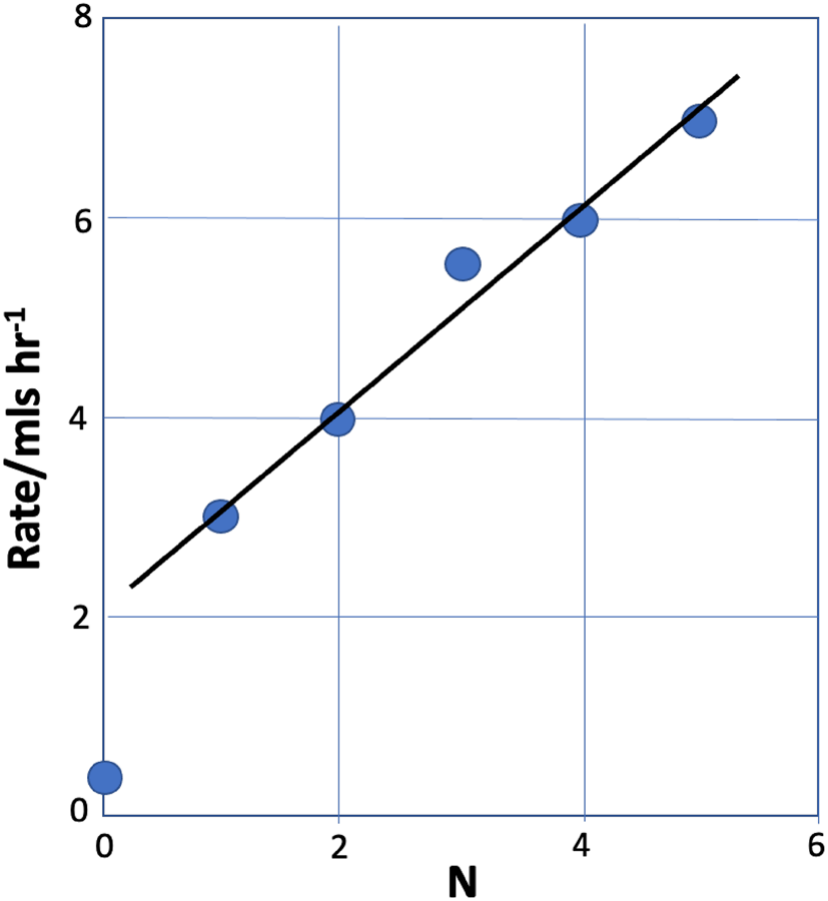
Rate of hydrogen evolution from alcohols as a function of carbon number* N* in the molecule [29]. *N* = 0 corresponds to CO, which is hydrogen produced solely from water in the photocatalytic water gas shift reaction

So—is it possible to find a sacrificial system where more of the hydrogen evolved comes from water splitting? Yes, it is; for instance, if the sacrificial is a series of polyols, then as the chain length increases (and hence the number of associated OH groups), so the rate of hydrogen evolution increases (Fig. 4) and the ratio of hydrogen derived from water itself increases (Table 1). The percentage asymptotically approaches 50% with increasing chain length [30]. The equation for this is as follows, and, effectively, when chain length* n* is large, then the ratio of hydrogen deriving from water to that from the alcohol approaches 1.

3 $${\text{C}}_{n} {\text{H}}_{{{2}n + {2}}} {\text{O}}_{n} + \, n{\text{H}}_{{2}} {\text{O}} \to n{\text{CO}}_{{2}} + \, \left( {{2}n + {1}} \right){\text{H}}_{{2}} .$$

**Table 1:** Moles of hydrogen per mole of alcohol during photo-reforming of various polyols and the percentage of total hydrogen evolved that is derived from water

Alcohol	Molar fraction	Percentage hydrogen from water
Methanol	3	33.3
Ethanol	5	40.0
Glycerol	7	42.8
Erythritol	9	44.4
Xylitol	11	45.5

A range of alcohols work for such photo-reforming, except that the stoichiometry generally changes to give 50% of the hydrogen evolved coming from water [31–33]. For instance, for n-alcohols, see Eq. 4 below—here the alkyl chain is evolved as the respective alkane.

4 $${\text{RCH}}_{{2}} {\text{OH }} + {\text{ H}}_{{2}} {\text{O}} \to {\text{CO}}_{{2}} + {\text{ RH }} + {\text{ 2H}}_{{2}} .$$

Even further—is it possible to find systems where all of the hydrogen evolved derived from water? Again, the answer is yes. For instance, if acetaldehyde is used as the sacrificial agent, then the stoichiometry is as follows [31, 32], the hydrogen is evolved is solely from water and the carbon chain is evolved as the respective alkane, just like the corresponding alcohol as described above. The difference is that all the hydrogen evolves from water.

5 $${\text{CH}}_{{3}} {\text{CHO }} + {\text{ H}}_{{2}} {\text{O}} \to {\text{CH}}_{{4}} + {\text{ CO}}_{{2}} + {\text{ H}}_{{2}} .$$

**Fig. 5:**
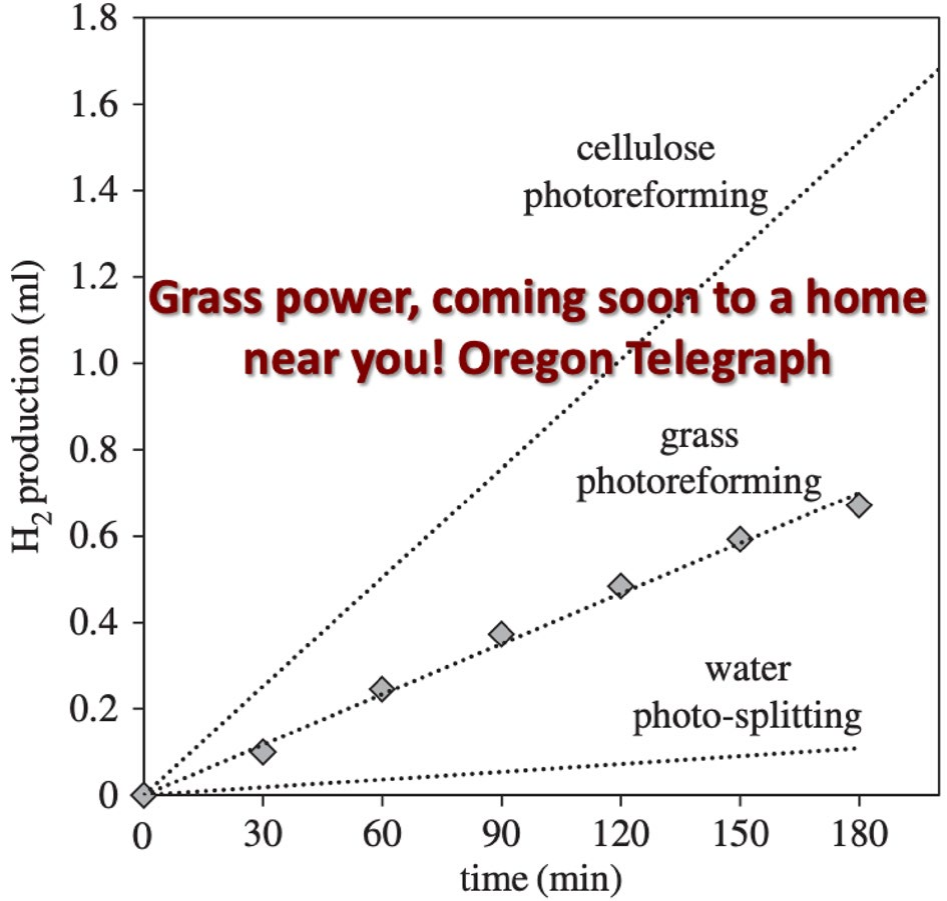
Hydrogen evolution from the photo-reforming of cellulose and grass using Pt/TiO_2_ catalysts; Adapted from Ref. [34] with permission from the Royal Society

As can be seen from the above, a wide variety of organic molecules can be used as hole scavengers, aldehydes and alcohols of various kinds [31–33], saccharides [30] and others, such as amines [30]. This even extends to polysaccharides that occur widely in nature, and that are themselves the subject of intense scrutiny regarding re-use and recycling from waste. These include cellulose and even raw biomass. We showed in 2016 [34] that not only solid cellulosic powder can be converted, with light and a Pt/TiO_2_ catalyst, into hydrogen, but that even grass could work, as seen below in Fig. 5, with the result that, as can be seen in the figure, rather over-the-top headlines appeared in a wide spectrum of publications!

Although a wide variety of organics can be used, not all can, since there are some basic rules that must be followed regarding the structure of the organic, which are:

1. it must have an active functional group (e.g., alcohol, carbonyl);
2. it must have a hydrogen alpha to that active group.

Hence, organics that are ineffective are those without the alpha hydrogen, and these include ketones and carboxylic acids [29–33].

### Mechanism of Reaction

**Fig. 6:**
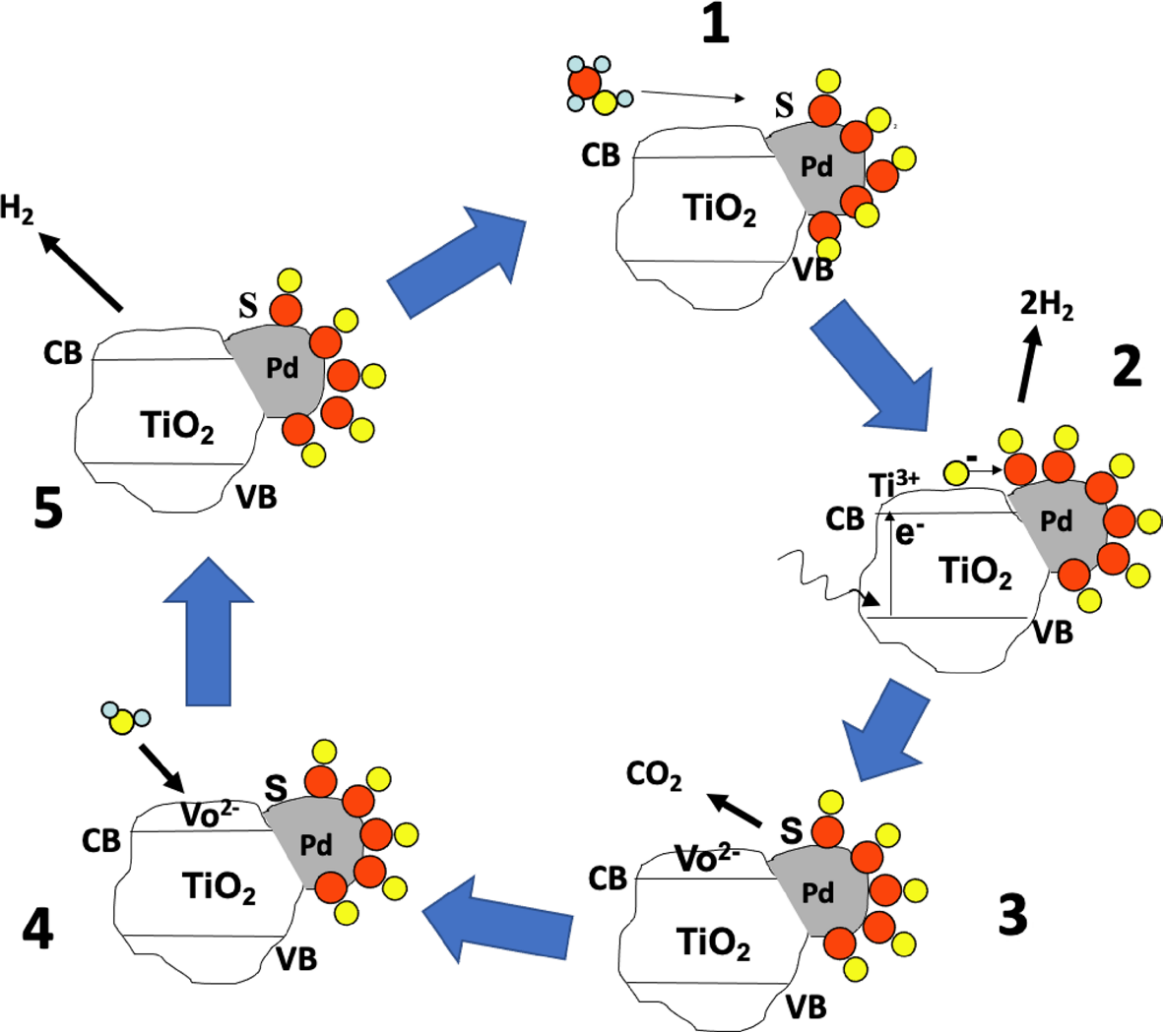
A pictorial scheme of the mechanism of photocatalytic conversion of methanol on Pd/TiO_2_.* S* Vacant site on the metal surface,* VB* valence band of the oxide,* CB* conduction band of the oxide, *V*_*o*_^*2–*^ anion vacancy in the titania lattice. Atoms:* red circles* carbon,* yellow circles* oxygen,* small blue circles* hydrogen. The steps are (*1*) methanol adsorption at a vacant site on the metal nanoparticle (NP), with dehydrogenation in (*2*) to give hydrogen into the gas phase and adsorbed CO on the metal. Also shown is electron–hole excitation by incoming light, to create the active surface species on the titania—the hole, *O*^–^. In (*3*), the hole has attacked the CO to give CO_2_ in the gas phase and leave a vacant site on the metal, and an anion vacancy on the oxide, which reacts with water in (*4*) and (*5*) to yield more hydrogen and to heal the anion vacancy at the surface. That completes the photocatalytic cycle

So, we need to ask ourselves, exactly how does this work, what is the role of the sacrificial agent and of the co-catalyst? If we turn to the literature, we can find various approaches to this, but our view is illustrated in Fig. 6. Common to all mechanistic views is the initial photoexcitation of the semiconductor oxide, in this case TiO_2_, by electron–hole excitation (Fig. 6, *2*). However, prior to this, and before light is admitted, we have the catalyst (Fig. 6, *1*), which can be either in a reduced state as metal NPs (by pre-reduction [35], or made by sol immobilisation for instance [36]), or as oxide (after the usual calcination and cooling in air), or can be photo-deposited by having the metal precursor in the solution with the photoactive support [37]. Depending upon the environment of the photocatalysis experiment, the surface may then be covered by adsorbate. In Fig. 6, *1* we show the example where the Pd surface is covered by an adsorbate, in this case CO groups, left adsorbed by dehydrogenation of the molecule, which has been shown to occur at ambient temperature on precious metals [38–41]. As mentioned, when the light is turned on, so the photocatalysis is initiated by electron–hole excitation to promote an electron from the valence band (VB) to the conduction band (CB), creating a hole (often, and somewhat confusingly, labelled as *h*^+^, whereas it is effectively *O*^–^). We originally proposed in 1999 [42] that the main role of the photoexcitation is to provide the hole to oxidise the adsorbate on the Pd [30, 33, 42, 43], with CO_2_ then released from the surface, which is otherwise blocked by strongly held CO (Fig. 6, *3*). This then leaves two sites on the surface of the catalyst: the vacant site on Pd left by the CO loss, and the anion vacancy on the TiO_2_ formed by CO_2_ evolution. The vacancy on the Pd is refilled by methanol in the liquid (or gas [30]) phase, while we contend that the highly reactive anion vacancy is filled by water reduction, producing hydrogen into the gas phase and completing the photocatalytic cycle (Fig. 6, *4* and *5*).

Photocatalytic processes need to be also chemically and electrically balanced, something that is relatively rarely reported in the literature, so the mechanism shown in Fig. 6 above can also be written as follows:

6 $${\text{CH}}_{{3}} {\text{OH }} + {\text{ S}}_{{{\text{Pd}}}} \to {\text{CO}}_{a} + {\text{ 2H}}_{{{2}g}} ,$$

7 $${\text{TiO}}_{{2}} + h\nu \to {\text{TiO}}^{ + } + {\text{ O}}^{-} ,$$

8 $${\text{CO}}_{a} + {\text{ O}}^{-} \to {\text{CO}}_{{{2}g}} + V_{o}^{-} + {\text{ S}}_{{{\text{Pd}}}} ,$$

9 $${\text{H}}_{{2}} {\text{O }} + V_{o}^{-} + {\text{ TiO}}^{ + } \to {\text{H}}_{{{2}g}} + {\text{ TiO}}_{{2}} ,$$

10 $${\text{CH}}_{{3}} {\text{OH }} + {\text{ H}}_{{2}} {\text{O }} + h\nu \to {\text{CO}}_{{{2}g}} + {\text{ 3H}}_{{{2}g}} ,$$

And similar balanced equations can be written for the other sacrificial agents described above. The 3:1 stoichiometry of the reaction was shown in the early papers [42, 44] and confirmed more recently by others [45].

Thus, in this mechanism, the metal plays a pivotal role in the whole process, by adsorbing and dehydrogenating/decarbonylating the incoming molecules, a property that Pd is known to possess [38–42, 44, 46, 47]. Others have provided different descriptions of the role of the metal. So, for instance, Joo et al. [48] propose that the role of the metal is merely to recombine hydrogen, which is produced on the titania, by reverse spillover, and which would otherwise be a slow step in the process. Others claim a kind of nano-electrical circuit in which H^+^ desorbs into the liquid phase and then interacts with OH^–^ to produce H_2_. However, it must be noted that the reaction proceeds well in the gas phase, and often better than in the liquid phase [28, 49], and it is difficult to envisage this mechanism being involved in that case. A further role proposed is that the presence of the metal traps the light-excited electron for a time and thereby extends the lifetime of the *e*–*h* pair, giving them more chance to react [50–52]. It may well be that this role is combined with the other mechanisms described above.

**Fig. 7:**
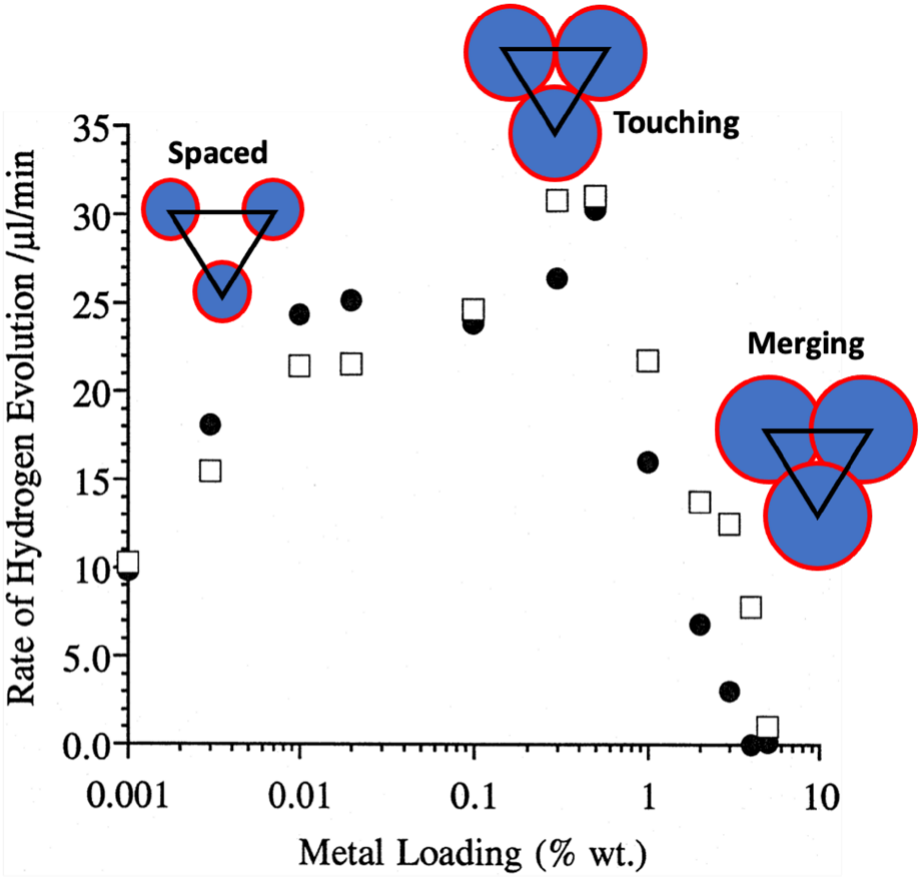
Dependence of hydrogen evolution rate upon weight loading. The arrangement of NPs on the surface is shown schematically. At low loading they are separate, at the maximum rate they are touching, and above that the particles begin to merge. Adapted from reference [42]

One of the notable features of these reactions is a very strong dependence of reactivity upon loading of the metal. Often it is found that very low loadings of metal are quite effective at promoting the reaction, see Fig. 7. The maximum rate here is at ~ 0.5–1% weight loading of Pd on the titania, but even as low as 0.01% is still quite effective, which is a useful property if these were used commercially, since precious metals, especially Pd at present, are extremely expensive. We will return to this point later when comparing the reactivity of other metals. This maximum is also reported for studies by other workers and for different metals. The maximum is shifted for some metals, but the maximum is generally at quite low loadings. So why is there this maximum?

In our original work on this subject, we proposed that the reason for this maximum was that the reaction took place at the interface between the metal and the support [44]. Geometrically, then, there is a relationship between the weight loading and the perimeter length, which has a maximum. However, this model produces a maximum at too high a loading of metal compared with experiment, assuming hemispheres, and is even still too high if we imagine that the particles are flat, circular monolayer islands. So, the model was modified to include an active perimeter that is somewhat remote from the particle edge [44], and has been refined recently to incorporate the area around the metal particles, and this is described in more detail in the section “The EPAO Kinetic Model” below.

### Variation with Metal

**Fig. 8:**
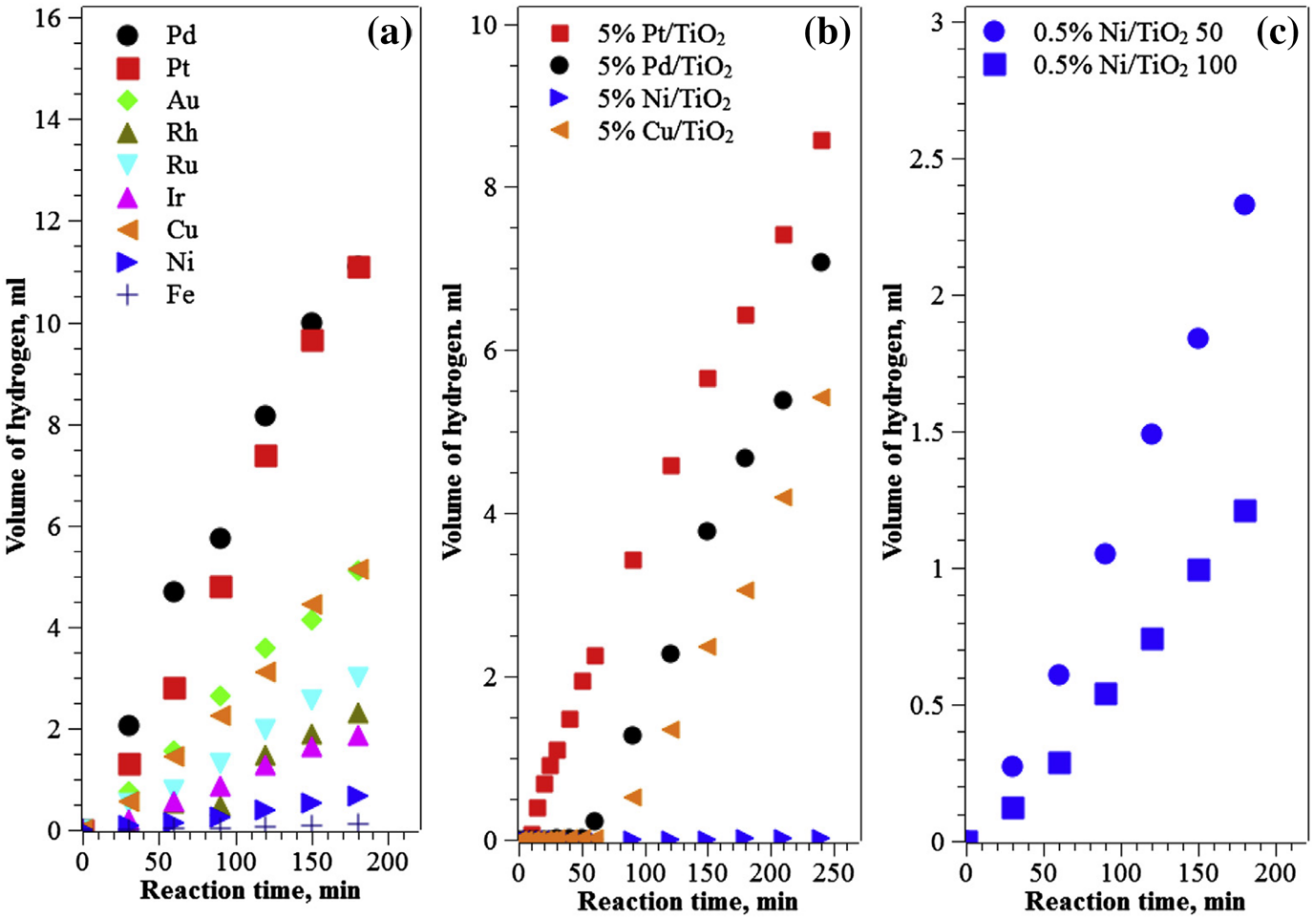
**a** Hydrogen evolution from 0.5% metal/TiO_2_ (P25) during solar simulator irradiation: **b** for 5% metal/TiO_2_ (P25): **c** 0.5% Ni/TiO_2_(P25) after external reduction at 350 °C, followed by passivation in air at two different temperatures. Courtesy of Elsevier from Ref. [35]

Most of the above dealt with photocatalytic reforming of methanol on Pd/TiO_2_ catalysts. Although Pt and Pd appear to be the most active for this reaction, other metals also show activity. This is shown in Fig. 8. The general trend seen here is that the most easily reduced metals tend to show the highest rates (Fig. 8a), and there is thus a relationship to the enthalpy of reduction of the oxide with methanol [35]. Figure 8b shows that rates are usually lower at high loadings of metal, and Fig. 8c shows that, at least to some degree, low rates are due to difficulty of reducing the metal from its oxide in situ. It also shows us that more earth-abundant metals can be used, even if they are not as yet the most efficient. Copper in particular shows good rates if prepared in the right way.

### Hydrogen Production—Conclusion

Here we have shown that a variety of organic molecules can be used to produce hydrogen. The types of oxygenate molecules that can be converted is wide-ranging and that range is governed by some simple rules—the molecule must have an active functional group, such as an alcohol or aldehyde function, coupled with an adjacent C–H bond. Thus, carboxylic acids and ketones are ineffective sacrificial agents for anaerobic photocatalysis. Such photocatalysis can be extended to other molecules, such as amines, and to bio-products such as cellulose and even biomass (grass) itself directly.

Here we have dealt with only TiO_2_ as a support, and its limitations are well known. Before we describe these, let us remind ourselves that it still the most versatile photoactive material around, due mainlyits relative cheapness, abundance, and photo-hydro stability in UV–visible light. Hence its use in many photo-related applications as outlined above in the “Introduction” to this section. The presence of certain metals as NPs on the surface generally enhances activity.

Nonetheless, this process does have a number of disadvantages. Although it is often very quantum efficient in the use of the photons it can absorb, that photon range is limited to that shorter than ~370 nm wavelength. So much work has been carried out with materials that have an absorption range extending into the visible, including doped titanias and materials such as carbon nitride. Good recent reviews of this kind of work are given elsewhere [53, 54]. The ultimate aim, of course, is to make hydrogen-producing systems that can split water directly, without the use of any sacrificial agents (thermodynamically extremely uphill), using a wide range of visible light, possibly with two photon absorption and with long-term stability and good quantum efficiency. So far this has not been achieved, though there are reports of demonstrator systems of this type in development [55].

Here, the focus has been on partial oxidation of organics for hydrogen production using anaerobic photo-reforming, but in what follows we will consider other aerobic oxidation routes including total oxidation.

## Photocatalytic Oxidation of Organic Pollutants

As we have seen in the previous section, the ultimate source of electrons, if not the hydrogen itself, in the photocatalytic production of H_2_ is usually an easily oxidised species that is present in the photosystem; the latter species is often referred to as a sacrificial electron donor (SED). A typical, general, simplified form of the reaction equation for the photocatalysed production of H_2_ by platinised TiO_2_, Pt/TiO_2_, is as follows:

11 $${\text{2H}}^{ + } + {\text{2SED}}\mathop{\longrightarrow}\limits_{{{\text{UV}}}}^{{ {\text{TiO}}_{{2}} /{\text{Pt}}}} {\text{H}}_{{2}} + {\text{2SED}}^{ + } ,$$

where SED^+^ is the oxidised form of the SED, which is usually then readily oxidised, eventually to mineral species, such as H_2_O, CO_2_ and mineral acids. Note that TiO_2_ is frequently ineffective in promoting this reaction in the absence of a co-catalyst, such as Pt, on its surface and, as a result, the enhancement factor, *δ*, for reaction (11) is at least very large, if not *∞*, given:

12 $$\delta = {\text{ rate of SPC with co-catalyst}}/{\text{rate of SPC without}}.$$

The photocatalysed oxidation of organic pollutants (PCO) is very similar to the photoproduction of H_2_ since, once again, an organic species, the ‘pollutant’, acts as the source of electrons but, in PCO, O_2_ rather than H^+^ is the target scavenging species for the photogenerated conduction band electrons. Although many different co-catalysts, for example Pt [56], Pd [57], Au [58] and Ag [59], have been used to promote PCO, Pt is usually the most effective and, not surprisingly therefore, the most well-studied. Because of the popularity of TiO_2_ as a photocatalyst and Pt as a co-catalyst, this brief overview of the role of metal NPs in promoting PCO is focussed on the use of TiO_2_ or TiO_2_/Pt, as is reflected by the following general reaction equation for PCO:

13 $${\text{O}}_{{2}} + {\text{pollutant}}\mathop{\longrightarrow}\limits_{{{\text{UV}}}}^{{{\text{TiO}}_{{2}} {\text{ or TiO}}_{{2}} /{\text{Pt}}}}{\text{minerals }}\left( {{\text{e}}.{\text{g}}.{\text{ H}}_{{2}} {\text{O}},{\text{ CO}}_{{2}} {\text{ and acids}}} \right).$$

Note that, in contrast to reaction (11), TiO_2_ alone is able to promote reaction (13) and, as a consequence, the values of the enhancement factor for PCO, reaction (13), are usually much more modest than they are for reaction (11) and usually fall in the range 0–8 and are most typically found to be between 2 and 4. It is a curious feature of PCO in general that the enhancement factor for PCO is found to be so similar and limited in value for many different organic test pollutants systems. This similarity suggests a common cause, namely a similar mechanism; a simple kinetic model based on such a mechanism is described in a subsequent section.

### The Role of Pt and the Effect of Loading on PCO Mediated by Pt/TiO_2_

It is possible to deposit the Pt onto TiO_2_ using a number of very different methods; however, the most popular methods continue to be photo deposition (PD) or thermal reduction (TR) under a stream of H_2_ [60, 61]. Both methods usually generate a final product comprising a homogeneous dispersion of Pt NPs, NPs, over the surface of the TiO_2_ [62, 63]. Often in studies of PCO using TiO_2_ with a Pt co-catalyst deposited by PD or TR, reaction (3), the maximum rate is observed for a wt% Pt(max) value of 0.5–1 wt%, at which, typically, the average radius of the Pt NPs is ca. 0.5–1 nm [62, 64]. Interestingly, as noted in the previous section, this feature is also exhibited in studies of reaction (11), which suggests a similar mechanistic origin.

The kinetics of the photocatalytic oxidation of organic pollutant by TiO_2_ are usually assumed to be determined by the rate of reduction of O_2_ by the photogenerated, conduction band electrons, *e*^–^,

14 $${\text{O}}_{{2}} + e^{-} \mathop{\longrightarrow}\limits^{{{\text{TiO}}_{{2}} {\text{ or TiO}}_{{2}} /{\text{Pt}}}} {\text{O}}_{{2}}^{-} ,$$

where, O_2_^–^ is superoxide, which can be reduced further to hydrogen peroxide and eventually water [56, 63]. This assumption appears reasonable provided the organic pollutant is present at a high concentration and/or is very easily oxidised and is likely to be satisfied in all cases where a SED, such as methanol and ethanol, is used as the test organic ‘pollutant’. On this basis it seems reasonable to assume the biggest values of *δ* will be found for reaction (13) when a SED is used as the ‘pollutant’ and this is demonstrated in a later section detailing reported *δ* values for different organic pollutants.

When reaction (14) is the rate-determining step (RDS) in PCO, then, in order to maintain the photo-oxidation process, reaction (13), it is necessary to avoid the accumulation of the photogenerated electrons, since this would increase the rate of electron–hole recombination and lower the quantum efficiency of the photocatalytic system. Not surprisingly, therefore, regardless of the choice or concentration of the test organic pollutant, the rate of PCO is usually found to be negligible in the absence of O_2_ and to increase with increasing concentration of O_2_ [13]. It follows that, under these circumstances, anything that can improve the rate of reaction (14) will produce an increase in the value of the overall photonic efficiency of the PCO process and so render *δ* > 1.

**Fig. 9:**
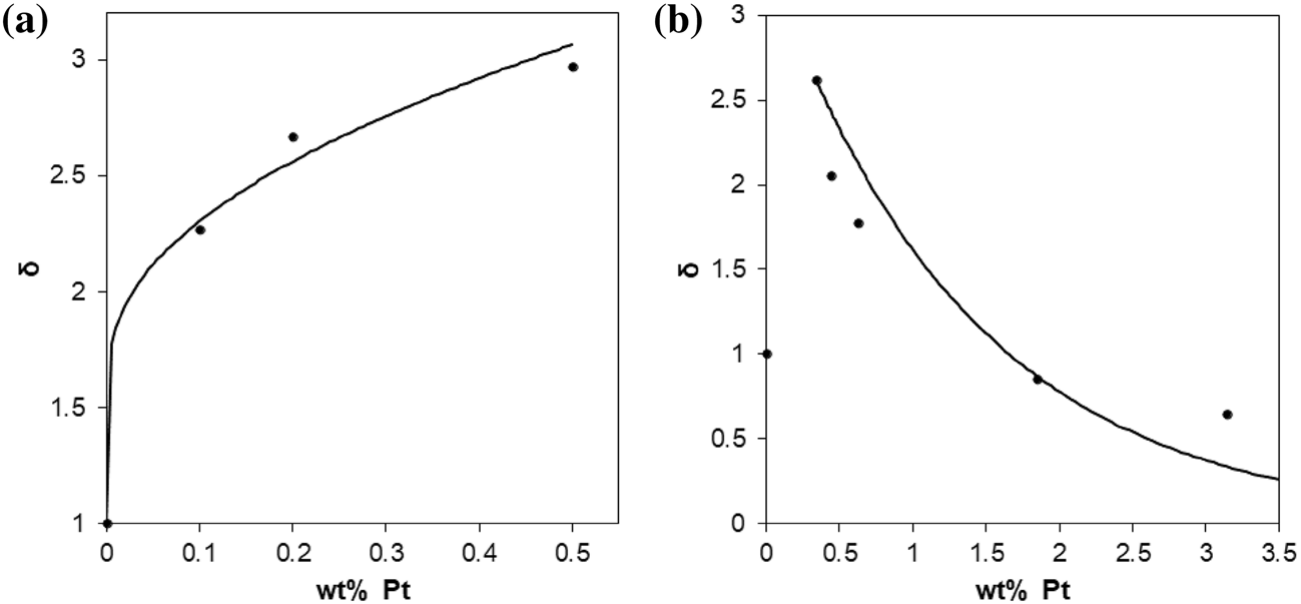
**a** Illustration of kfA with the plot of enhancement in rate enhancement factor, *δ*, vs wt% Pt for Hombikat 100 (5 g/L; anatase) TiO_2_ in the photocatalysed oxidation of dichloroacetic acid (DCA) (1 mM), pH 3, 10 mM KNO_3_, O_2_ saturated [63]. **b** Illustration of kfB with the plot in *δ* vs wt% Pt for the photo-oxidative bleaching of rhodamine B (12 mg dm^–3^) by P25 TiO_2_ (2 mg mL^–1^).* Solid lines* EPAO (expanding photocatalytic area and overlap) kinetic model fits to the data [56]

Numerous lifetime studies show that Pt NPs can act as a sink for the conduction band electrons photogenerated in the TiO_2_ and also then mediate their transfer to O_2_, i.e., catalyse reaction (14) [65, 66]. It is no surprise therefore that Pt NPs on TiO_2_ often enhance the rate of reaction (13), although perhaps it is slightly surprising that the enhancement factor is often quite modest, ca. 2–4 times, as noted previously. In particular, it is usually found that, at low loadings (0 to ca. 1 wt% Pt), the PCO rate increases with increasing wt% Pt (henceforth referred to as kinetic feature A, kfA). However, less obviously, additional studies of reaction (3) suggest that, above a threshold wt% Pt value, wt% Pt (max), the rate then decreases with increasing Pt loading; this effect is henceforth referred to as kinetic feature B, kfB) [56, 66]. Perhaps most surprisingly, given the well-known nature of this effect, an inspection of the literature reveals no report of a rate versus wt% Pt profile for reaction (13), which clearly shows these two very different kinetic features, kfA and kfB, at low and high wt%Pt levels, respectively. Instead, as we shall see, most reports focus on using just one wt% Pt loading, usually 0.5–1 wt% to highlight the usual modest (2- to 4-fold) enhancement in rate. However, there are some clear examples of these two individual kinetic features for reaction (13) in the literature, as illustrated by the plots in Fig. 9. Thus, Fig. 9a illustrates the first of these features, kfA, in the form of an increase in enhancement factor, *δ*, versus wt% Pt, for the photocatalysed oxidation of dichloroacetic acid (DCA), sensitised by anatase TiO_2_ (Hombikat 100) in aqueous solution [63]. Further work on this system showed that, after the calculated value of *δ* peaks at ca. 0.5 wt% Pt, it then decreases by 17% by 1 wt% Pt, thereby starting to exhibit the second (after the threshold) kinetic feature, kfB. Figure 9b provides a much better illustration of this second kinetic feature of reaction (3), kfB, but for a different photocatalytic system, namely the photo-oxidative bleaching of rhodamine B by P25 TiO_2_ [56]. Note that, in the latter plot, the enhancement factor drops below that of naked TiO_2_, when *δ* = 1, and appears to tend to zero at high wt% Pt. Thus, any kinetic model developed to explain the observed kinetics for PCO must be able to embrace the usual variation of *δ* versus wt% Pt and its kinetic features, kfA and kfB.

As noted previously, the above two kinetic features of PCO and reaction (3), kfA and kfB, are also often observed in the photocatalysed production of H_2_, reaction (11). The similarity in the kinetic features associated with rate versus wt% Pt for reactions (11) and (13) is perhaps not too surprising giventhat both involve the oxidation of an organic species, namely, a SED/test pollutant, with only the nature of the scavenger of photogenerated electrons, H^+^ or O_2_, being different. In the previous section, a simple kinetic model, the expanding photocatalytic area and overlap (EPAO) kinetic model, was mentioned as one that provides a rationale and good fit to the kinetic features associated with the photocatalysed reduction of water by Pt/TiO_2_, reaction (11). In the next section, this same model is used to interpret the similar features exhibited by PCO and reaction (13), as illustrated in Fig. 9a and b.

### The EPAO Kinetic Model

As noted above, and illustrated by the example data in in Fig. 9a,b, in the photocatalysed oxidation of organic pollutants by TiO_2_/Pt, the rate of reaction (*r*(PCO)),—and thus the enhancement factor, *δ*—is often found to increase with wt% Pt at low loading, kfA, and, after reaching a maximum value at wt% Pt(max), decrease with increasing wt% Pt, kfB [56, 66]. In the literature, the first of these kinetic features, kfA, is usually associated with an increase in the scavenging of the photogenerated electrons with wt% Pt [67–69], i.e. enhanced charge separation, due presumably to an increase in the area and/or number of the Pt NPs, *N* [56]; although note, however, that in practice it appears that *N* is approximately constant with increasing wt% Pt for TR- and PD-produced NPs. Other explanations for the enhancement in rate with increasing wt% Pt include improved light absorption by surface plasmon resonance [70] or doping [72, 74], and/or better O_2_ absorption [73–76]. However, few have attempted to address the often-observed variation of rate with metal (usually Pt) loading, which peaks at ca. 1 wt% and then decreases significantly.

Whilst the first of the kinetic features, in a rate versus wt% Pt plot, kfA, appears obvious, and easy to rationalise, as outlined above, the second, kfB, illustrated in Fig. 9b for the photocatalysed oxidation of rhodamine B by P25 TiO_2_/Pt, does not. It has been suggested recently that kfB is due to the diffusion-limiting transport of O_2_ to the Pt NPs when there is too much Pt on the surface, so that electron–hole combination at the Pt NPs dominates [57]. However, the sharpness of the transition from increasing (kfA) to decreasing (kfB) rate (or *δ*) value suggests that this is unlikely to be a primary cause. Another, commonly invoked explanation for kfB is that, at these high loadings, the Pt NPs absorb and block out the light reaching the TiO_2_ particles [77], but this ‘UV-screening’ hypothesis is also easily shown to be unlikely. For example, in the TiO_2_/Pt system used to generate the data illustrated in Fig. 9b, we know that the average particle size is ca. 1.4 nm at wt% Pt(max) = 0.34%. Assuming for the system that the Pt NPs are in the form of a uniform dispersion of hemi-spherical spheres on the P25 TiO_2_ particles, it can be calculated that the value of *N* will be 4.4 × 10^15^ particles m^−2^. If we assume this value of *N* does not change with increasing wt% Pt, as is often found to be the case, it can be shown that only 6% of the surface would be covered, i.e. UV-screened, when the wt% Pt reached a value of 3.1 wt% Pt; thus, UV screening at 3.1 wt% Pt would be negligible. However, from the data illustrated in Fig. 9b, for this system, the observed rate of PCO at 3.1 wt% Pt is well below even that of non-platinised P25 TiO_2_. Clearly, based on the above calculations, for this P25 TiO_2_/Pt—rhodamine B photocatalytic system, the significant loss in rate of PCO exhibited at 3.1 wt% Pt cannot be due to UV-screening by the Pt NPs [56].

Finally, strong metal substrate interaction (SMSI) effects have been invoked to provide a rationale for the observed increase [78] and decrease in rate phases [79–81]. However, such effects have been ascribed largely to photocatalysts that have been subjected to vacuum or reducing conditions, thereby allowing the surface TiO_2_ to be reduced and rendered sufficiently mobile that they can coat the surface of the Pt NPs, rendering them non-catalytic [81]. However, these preparation conditions do not apply to most examples of TiO_2_/Pt photocatalysts.

Here, we suggest there may be another, simple, explanation for the two kinetics effects often exhibited in a rate versus wt% Pt plot. Indeed, the strong similarity between the rate versus wt% Pt profiles reported for reactions (11) and (13) suggests a common cause/mechanism. As a result, the same EPAO kinetic model that has been used recently [62] to provide a rational for reaction (11) may be used as such for PCO and reaction (13). The key assumptions in an EPAO model for PCO are as follows:

1. Regardless of the value of the wt% metal, the metal is distributed uniformly as a fixed number, *N*, of hemispherical NP islands of radius, *r*, across the surface of the semiconductor photocatalyst particles, each spaced a distance, *R*, apart, where the distance depends upon their packing (hexagonal or square packed).
2. The rate of PCO, reaction (13) (*r*(PCO))*,* is proportional to the product of the total photocatalytically active area, *A*_T_*’*, per square metre of photocatalyst, and its associated enhancement factor *δ*_EPAO_, plus that area that has not been activated, *A*_F_. It follows that at zero wt% metal the rate will not be zero and *δ* = 1.
3. The circular region of activation (RoA) surrounding each metal NP, has a radius *r*_z_ where *r*_z_ is a simple linear function of *r*, the radius of the metal island, the value of which depends only on the value of the wt% metal.
4. The value of *r*(PCO) inside the RoA is uniform and greater than that outside the RoA. Thus, the enhancement factor inside the *RoA* (*δ*_EPAO_) is > 1. As a result, the overall observed value of the enhancement factor, *δ*, exhibited by the system increases with increasing wt% metal, up to a threshold value, wt% metal(max), at which point the RoAs touch and *r*_z_ = *R*/2 = *r*_touch_.
5. The increase in *r*(PCO) with wt% metal is due to the appropriate increase in *r*_z_ and so *A*_T_*’* and is responsible for kfA.
6. When *r*(PCO) is at a maximum (*r*(PCO)_max_) wt% metal = wt%(max) and *A*_T_*’* = *A*_T_*’*(max). The photocatalytic area does not increase further, with any further increase in wt% metal and *r*, although a deactivating zone, due to overlap, does.
7. The sudden and striking decrease in *r*(PCO) above the wt% metal associated with *R*/2, i.e. above wt%(max), where *r*_z_ > *r*_touch_, is due the overlap of the expanding deactivation zones with the photocatalytically active area, A_T_*’*(max) and is responsible for kfB.
8. In areas of overlap, electron–hole recombination dominates to such an extent that no photocatalysis occurs and consequently, the overall value of *r*(PCO), and so *δ*, tends to zero with increasing wt% metal above wt%(max), as observed in kfB.

**Fig. 10:**
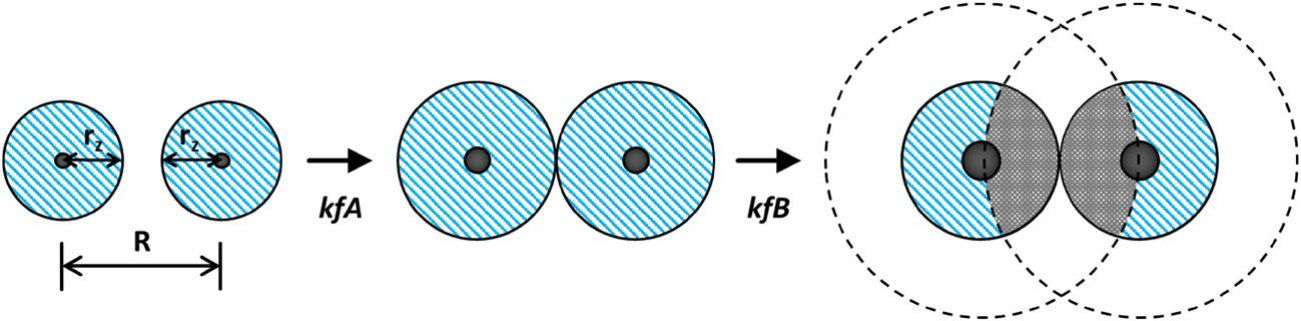
Schematic illustration of the key features of the EPAO model for photocatalysed oxidation of organic pollutants (PCO), comprising a pair of metal particles (black dots) radius *r*, on the surface of a semiconductor photocatalyst, with zones of photocatalytic activity* (blue hatched*) areas of radius *r*_*z*_, separated by a distance, *R*, which is determined by the number of particles, *N* and the type of packing (hexagonal or cubic). With increasing wt% the regions of activation, *RoA*s, increase and so do the values of *r*(PCO) and *δ*, as in kfA. Eventually, the *RoA*s touch and *r*(PCO) is maximal. After this point, as the wt% is increased further the *RoA*s overlap to an increasing extent and the values of *r*(PCO) and *δ* decrease, tending to zero, as in kfB

A schematic illustration of the key features associated with the EPAO model is given in Fig. 10.

Given the above assumptions, it follows that

15 $$r\left( {{\text{PCO}}} \right) = k(d_{{{\text{EPAO}}}} .A^{\prime}_{T} + A_{F} ),$$

where, *k* is a proportionality constant and *A*_*F*_ = (1 – *N*π*r*_z_^2^).

**Fig. 11:**
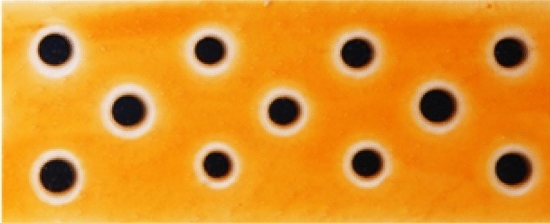
Photograph of an anatase, sol–gel TiO_2_ film, covered in Pt macro dots (*r* = ca. 1 mm), stained with AO7 and irradiated with UV radiation for 4 h. Reprinted with permission from [82]. Copyright 2020 ACS

In the growing rate stage, kfA,

16 $$A^{\prime}_{T} = N.\pi \left( {r_{z}^{{2}} {-}r^{2} } \right),$$

where the value for *N* is calculated from a knowledge of a paired data set of wt% of metal and metal particle radius, *r*, determined for the specific PCO system under study, usually using SEM. Once a value for *N* has been determined, it can then be used to calculate a value for *R*, the distance between metal sites, depending on whether the sites are hexagonal *R* = {2/(*N*.√3)}^0.5^, or square packed (*R* = (1/*N*)^0.5^. Knowledge of the value of *N* allows calculation of the variation of the metal particle radius, *r*, with wt% metal. In the initial increasing r(PCO) stage, kfA, it is assumed that the value of *r*_z_ = *r*_z_(incrs), is related directly to *r* by the following simple expression:

17 $$r_{z} \left( {{\text{incrs}}} \right) = \, a + br,$$

where *a* and *b* are constants. Eventually the activation zones, which expand with increasing wt% metal, and thus with increasing value of *r*, touch, as illustrated in Fig. 11, at which point *r*(PCO) is a maximum, as is *A*_T_′,* A*_T_′ (max) and *r*_z_ = *R*/2 = *r*_touch_ and *r* = *r*(max). It is the above part of the EPAO model that has been used to generate the solid line fit to the kfA data illustrated in Fig. 9a for the PCO of DCA by TiO_2_/Pt.

In the EPAO model, the subsequent decrease in rate with increasing wt% metal above the maximum, is due to the decrease in *A*_T_′ from its peak value of *A*_T_′ (max), as the extended zones of the neighbouring metal islands overlap and become deactivation zones, as illustrated by the grey zones in Fig. 10. In this work it is assumed that *r*_z_(decrs) is described by the expression:

18 $$r_{z} \left( {{\text{decrs}}} \right) = R/{2} + b^{\prime}\left( {r \, {-}r\left( {{\text{max}}} \right)} \right),$$

where *b*′ is a constant. It follows from the above, that in the decreasing *r*(PCO) with metal wt% stage, i.e. in the kfB zone, the value of *A*_T_′ is given by:

19 $$A^{\prime}_{T} = \pi N\left( {r_{{{\text{touch}}}}^{{2}} {-}{\text{ area of overlap}}} \right),$$

and this can be used, via Eq. (15), to model the decrease in rate as a function of wt% metal above the maximum rate. It is this part of the EPAO model that has been used to generate the solid line fit to the kfB data illustrated in Fig. 9b for the PCO of rhodamine B by TiO_2_/Pt.

### Direct Evidence for the EPAO model: Ring Photocatalysis

In a recent study of the photocatalytic oxidation of soot and acid orange 7, AO7, adsorbed onto the surface of a TiO_2_ film covered with macro-sized, photodeposited islands of Pt (*r* ≥ 1 mm), the appearance of RoAs, as assumed in the EPAO kinetic model, were readily observed [82]. A striking illustration of this effect is given in Fig. 11, namely, a photograph of an anatase, sol–gel TiO_2_ film, covered in Pt macro dots (*r* = ca. 1 mm), stained with AO7 and subsequently irradiated with UV radiation for 4 h. In this figure, the RoAs reveal themselves as white (bleached) circular zones surrounding each Pt macro island.

**Fig. 12:**
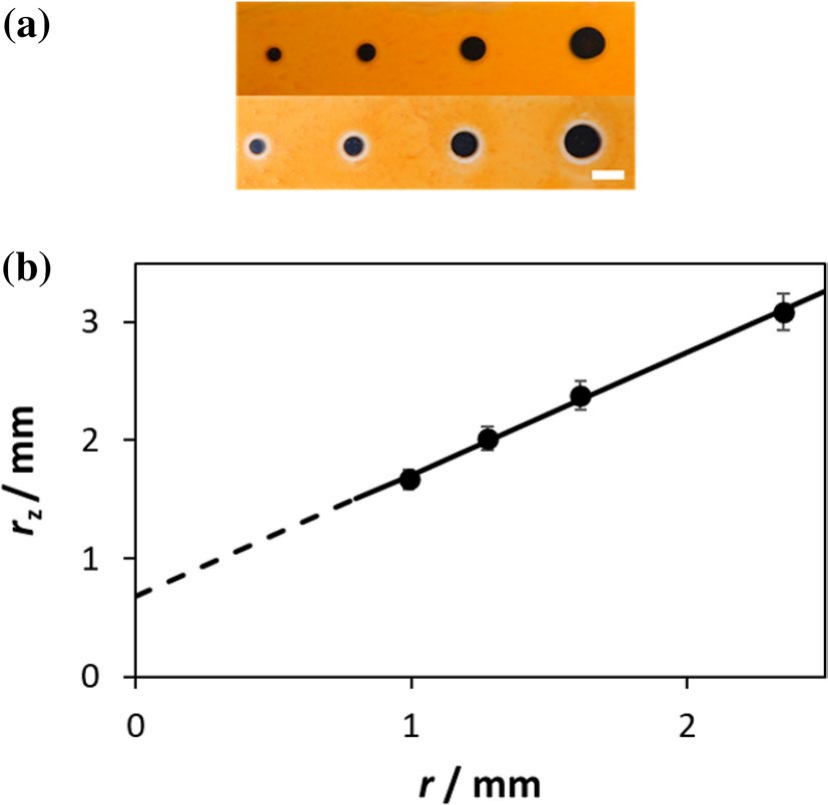
**a** Photograph of a sol–gel TiO_2_ film, with four Pt macro dots of different size, stained with AO7, before (*top*) and after (*bottom*) irradiated with UV radiation for 4 h;* scale bar* 5 mm. **b** Plot of *r*_z_vs * r* values derived from the photographs illustrated in **a**. Reprinted with permission from [82]. Copyright 2020 ACS

Further work on the same system shows that the radius of these rings of activation, *r*_z_, is related to the radius of the Pt ‘dot’, *r*, via an equation that is identical to Eq. (17) of the EPAO model. A rather nice illustration of this is given in Fig. 12, which shows a photograph of the same photocatalytic film as used in Fig. 11, but with different sized Pt ‘dots’, which clearly generate different sized rings of activation, see Fig. 12a. The subsequent plot of *r*_z_ vs *r*, generated using the images in Fig. 12a, is illustrated in Fig. 12b and is of the same form as that of Eq. (17) of the EPAO model.

This study also shows that, within the RoAs, the rate of destruction of the ‘pollutant’ (in this case AO7 or soot) is uniform and enhanced, typically by a factor of 3–5, compared with that area outside the RoAs. The latter kinetic feature forms an intrinsic part of the EPAO model and, although it is not clear what exactly determines the value of the enhancement value inside a RoA, *δ*_EPAO_, it appears reasonable to assume the more easily oxidised the pollutant, the greater value of *δ*_EPAO_.

The collection of macro-scale kinetic features exhibited by TiO_2_/Pt films described above is referred to, for brevity, here and elsewhere as macro ‘ring photocatalysis’ [82]. These features include (1) RoAs with a radius related directly to the Pt particle radius and (2) unform enhanced photocatalytic activity inside the RoAs. These features are also an inherent part of the EPAO kinetic model used to fit the observed kinetics features, kfA and kfB, as illustrated in Fig. 10. Thus, the results illustrated in Figs. 11 and 12 appear to provide direct evidence for the EPAO kinetic model, on a macro scale at least. But, it has to be said that, at present, there is as yet no direct evidence that ring photocatalysis applies to the Pt NPs on TiO_2_/Pt films and powders, although the concept is still relatively new and these are early days. Thus, the only evidence that ring photocatalysts applies to Pt NPs on TiO_2_ is indirect in nature and in the form of the often-observed variation in *r*(PCO) as a function of wt% Pt, and the associated kinetic features, kfA and kfB, and the good fit to the data, such as illustrated in Fig. 9, provided by the EPAO kinetic model.

### Theoretic Rationale for Ring Photocatalysis and the EPAO Model

**Fig. 13:**
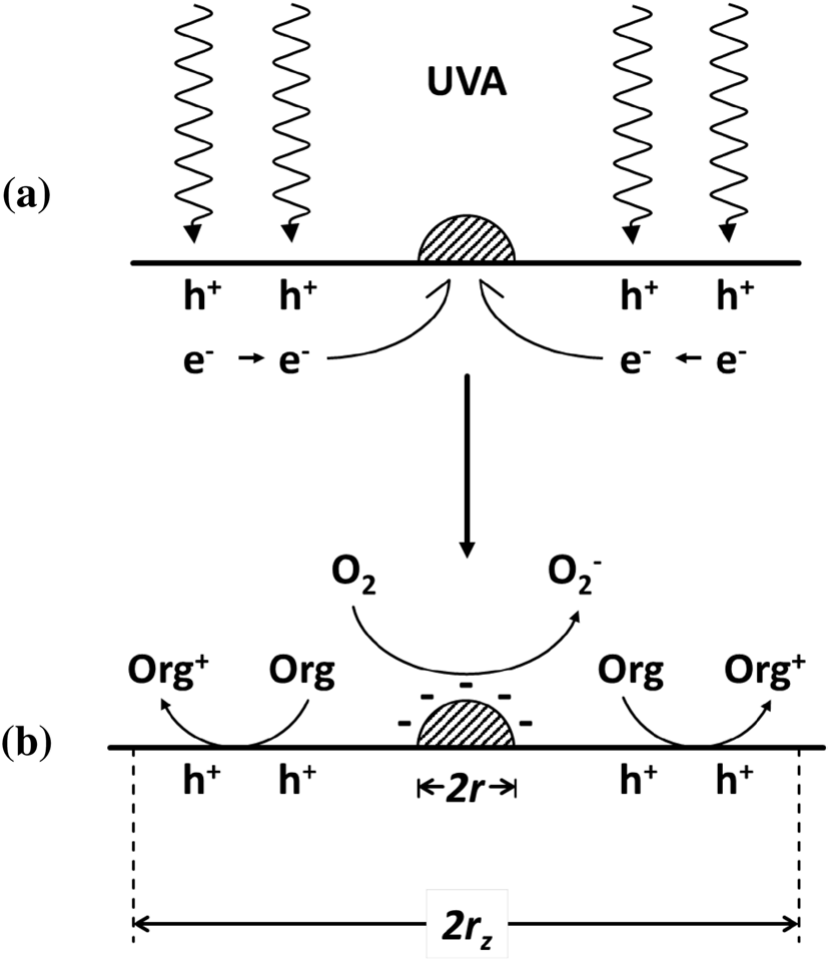
Schematic illustration of **a** the harvesting of conduction band electrons photogenerated on TiO_2_ by Pt particles and **b** their subsequent catalysed reaction with O_2_ and reaction of the photogenerated holes, *h*^+^, with the adsorbed organic pollutant. Adapted with permission from [82]. Copyright 2020 ACS

The EPAO model, which is applied here to PCO, but has also been applied to water reduction and reaction (11), is based on the concept of an extended reaction area around the Pt particles (macro and NP) Pt on the surface of the TiO_2_ photocatalyst. This appears a reasonable assumption if, as is often reported, the Pt metal islands aid the separation of the photogenerated electron–hole pairs by acting as (1) a sink for photogenerated electrons and (2) a catalyst for their subsequent reduction reaction with O_2_, in PCO. The electron sink role of Pt NPs, assumed by so many [56, 63] and here in the EPAO model, has been rationalised previously by others in terms of either a metallised semiconductor model, in which the Pt forms an ohmic junction with TiO_2_ [83] or one in which an accumulation layer is formed at the TiO_2_/Pt interface [84]. Whatever the cause, the effect will be to produce an electric field around the Pt particles which draws the conductance band electrons, photogenerated in the surrounding TiO_2_, to the nearest Pt particle. As a consequence, the RDS for reaction (13), the reduction of O_2_ via reaction (14) is enhanced, with an concomitant increase in both the overall rate and enhancement factor, producing values > 1. A schematic illustration of this Pt particle enhanced rate model is illustrated in Fig. 13 and is very similar to that proposed by Gerischer et al. [84] for Pt-island coated TiO_2_ particles for water reduction. The loss of activity, above a threshold wt% Pt, is due to the overlap of the Pt-induced electric fields, which create the RoAs, which causes significant electron–hole recombination in the regions of overlap and the rapid total loss of photocatalytic activity at wt% Pt levels that would otherwise be considered surprisingly low.

For photocatalytic systems in which the RDS for reaction (13) is not reaction (14), then the presence of Pt NPs might be expected to have little or no effect on the rate, so that *r*(PCO), appeared independent of wt% Pt. The latter situation might be expected to occur if the organic pollutant in reaction (13) is refractory and/or the oxidised intermediates, produced by its direct or indirect oxidation by a photogenerated hole, *h*^+^, are long-lived and readily adsorbed on the surface and so prone to reduction by the photogenerated electrons, thereby short-circuiting the overall photocatalytic process, reaction (13). Under such circumstances the actual rate would be low but *δ* would appear to ca. 1 at all wt% Pt values, and is referred to as an example of ‘unaffected’ kinetics.

Alternatively, as has been suggested by others [56], under these circumstances the Pt NPs might provide an additional route for photogenerated electrons and holes to recombine, via the following Pt-catalysed process:

20 $$h^{ + } + e^{-} \mathop{\longrightarrow}\limits^{{{\text{Pt}}\;{\text{NP}}}} {\text{heat}}.$$

In the latter case, no enhancement in *δ* might be seen but, rather than appearing to be independent of wt% Pt, the value of *δ* decreases with increasing wt% Pt [85, 86], and so, under such circumstances, the kinetics would appear ‘inhibited’ by the presence of Pt NPs.

**Fig. 14:**
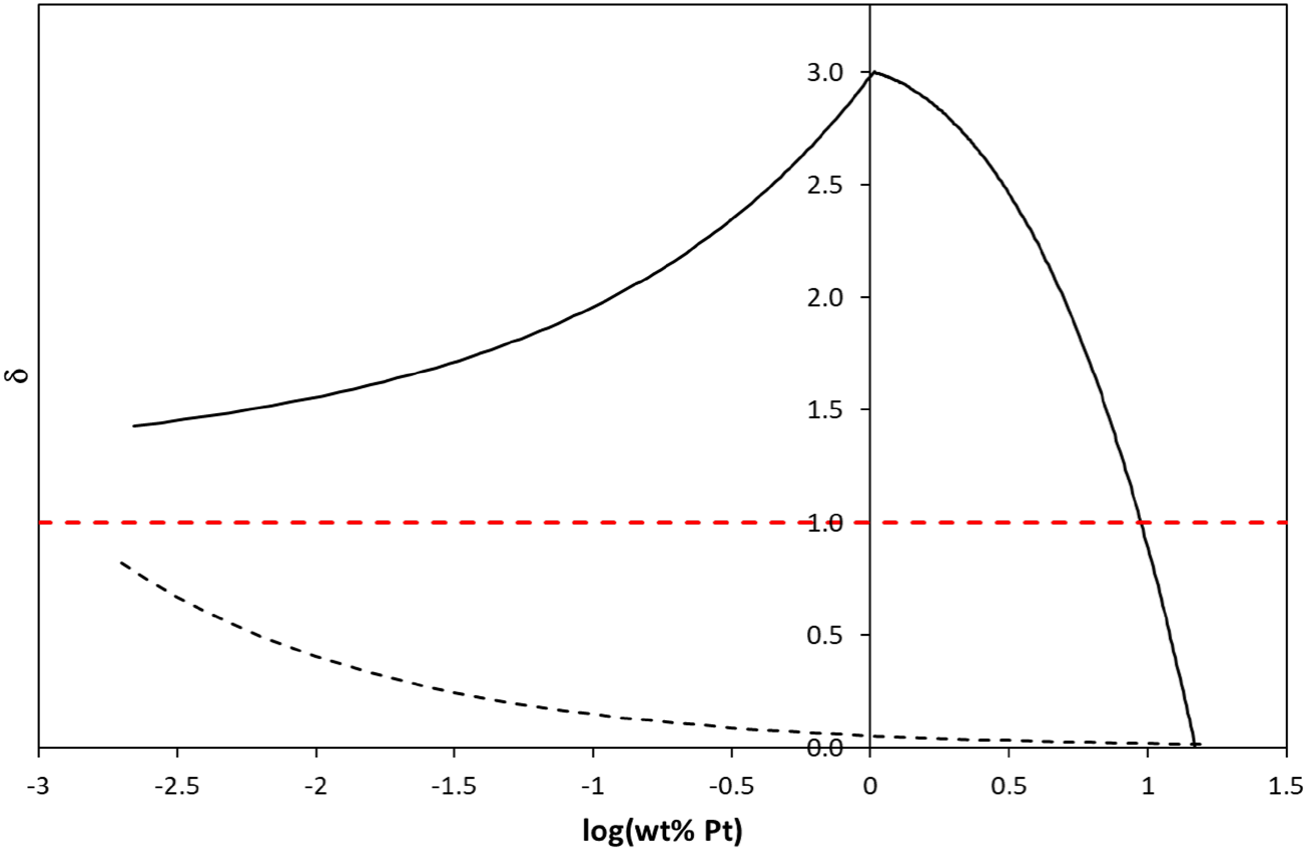
Schematic illustration of three possible *δ* vs log (wt% Pt) profiles for reaction (13) in which the PtNPs effect, enhanced (EPAO model-type,* solid line*), zero-change (*red broken line*) or inhibited (*black, broken line*) PCO kinetics

Lying between these two extreme *δ* versus wt% Pt profiles, the EPAO model provides a rationale for the more usually observed variation in *δ* versus wt% Pt, namely, (1) initially increasing *δ* with wt% Pt until reaching a maximum value, kfA, followed by (2) decreasing *δ* with wt% Pt until reaching a zero rate, well below that required to screen out all the incident UV light falling on the surface of the TiO_2_, kfB. A schematic illustration of examples of the *δ* versus log(wt% Pt) profiles associated with these three different scenarios, where PtNPs effect either, enhanced, zero-change or inhibited PCO kinetics, is illustrated in Fig. 14.

### Examples of Pt NP-Effected Enhanced, Zero-Change and Inhibited PCO Kinetics

As noted previously, given the significant interest in PCO, and the many studies of reaction (13), it is surprising that there appear to be no detailed studies of *δ* versus wt% Pt for such systems, unlike reaction (11). Instead, as noted earlier, most reports on PCO focus on using a fixed wt% of Pt, usually 0.5–1 wt%, to compare and contrast the usually improved efficacy of Pt NPs on PCO and reaction (13).

In a previous review on the effect of Pt on PCO [87] we showed that the rate of PCO, and so the value of *δ*, depends upon many parameters other than the wt% of metal and type of metal, including (1) method of metal deposition, (2) nature and physical form of pollutant, (3) nature and physical form of the semiconductor photocatalyst and (4) the experimental conditions employed. This vast array of variables makes any meaningful comparison of PCO rates and *δ* values difficult, if not impossible. Even when this is limited to just one semiconductor and metal, e.g., P25 TiO_2_ and Pt, the possible variations remain significant, and this is reflected in the large number of reports on even this ‘simple’ system. Table 2 summarises the major variables associated with PCO by TiO_2_ or TiO_2_/Pt photocatalysts.

**Table 2:** Major variables in photocatalysed oxidation of organic pollutants (PCO) using a TiO_2_ or TiO_2_/Pt photocatalysts. *ALD* atomic layer deposition,* DCA* dichloroacetic acid,* PD* photodeposition,* TR* thermal reduction,* UV* ultraviolet

Variables	Comments
Pt deposition method	
PD, TR, sputtering, ALD, chemical reduction and physical mixing, particle and/or cluster (of particles) size	PD and TR are the most popular methods of deposition. These methods have been reviewed recently [85]
The pollutant	
Organic or inorganic, ease of oxidation/redox potential, concentration, volatile (air-purification), dissolved in solution (water purification), or solid (self-cleaning films)	Most organic and inorganic pollutants undergo PCO. CO appears to be particularly difficult to oxidise using just TiO_2_ and much less so by TiO_2_/Pt, thus *δ* is > > 1 [88, 89]
The TiO_2_	
Anatase and/or rutile, method of preparation, film or powder, specific surface area, aggregated particle size (when dispersed in solution)	Some [65] have reported a much more striking enhancement in PCO rate (for DCA) with platinisation for rutile than anatase and ascribed it to its significantly lower conduction band redox potential, i.e. − 0.11 V cf. − 0.32 V vs NHE at pH 0, respectively [62]. Others have found the PCO (of phenol) activities exhibited by rutile and anatase are enhanced to a similar extent by platinisation [91]
Experimental conditions	
[O_2_], pH, ionic strength, temperature, humidity, rate of stirring or flow, size, shape, and composition of photoreactor, UV irradiance value, emission spectrum of UV source	Note that pH has a striking effect on rate if the pollutant is charged and forms a strong ion-pair with the oppositely charged surface of the TiO_2_ [92, 93]

In an attempt to limit the number of variables, and provide some focus on the effect on Pt on *δ*, Tables 3 and 4 have been constructed of reports on the use of P25 TiO_2_ only as the photocatalyst and Pt as a the co-catalyst, in the oxidation of organic pollutants either in solution (dispersed as a powder) (Table 3 [56, 61, 90, 94–101]), or in the gas phase, volatile organic compounds (VOCs) (Table 4 [55, 87, 91, 94–106]).

**Table 3:** Reported examples PCO systems and their *δ* values for the purification of water by P25 TiO_2_

Deposition method	wt% Pt	Pollutants (s)	*δ*	References
PD	1	MeOH (pH 5.1)	7.8	[94]
PD	1	EtOH (pH 5.1)	4.2	[94]
PD	1	DCA	3	[95]
ALD	0.34–3.1	RhB	2.6–0.5	[56]
TR	1	EtOH (pH 10.9)	2.4	[94]
PD	1	MeOH (pH 10.9)	2.4	[94]
PD	1	RhB	2.3	[96]
CR	3	MB	2	[97]
ALD	0.34–3.1	AB9	1.7–0.7	[56]
PD	0.1	Rh6G	1	[98]
Pt colloid precipitation [54]	1	2,4-DCPAA	1	[99]
PD	0.1	NG	0.8	[98]
TR	0.1–1.5	Phenol	1–0.6	[86]
PD and CR (Zn)	1	TCE	< 1	[100]
PD	0.15	DCA	0.8	[90]
PD	1	Chloroform (pH 5.4)	0.6	[94]
TR	1	CBA	0.4	[101]
PD	1	Chloroform (pH 5.6)	0.3	[94]
PD	1	TCE (pH 5.2)	0.1	[94]

*PD* photocatalytic deposition, *TR* thermal reduction, *ALD* atomic layer deposition, *CR* chemical reduction, *MeOH* methanol, *TCE* trichloroethylene, *BTEX* benzene, toluene, ethylbenzene and xylene, *EtOH* ethanol, *DCA* dichloroacetic acid, *RhB* Rhodamine B, *MB* methylene blue, *AB9* Acid Blue9, *Rh6G* rhodamine 6G, *2,4-DCPAA* dichlorophenoxy acetic acid, *NG* nitroglycerine, *CBA* chlorobenzioc acid

**Table 4:** Reported examples PCO systems and their *δ* values for the purification of water by P25 TiO_2_

Deposition method	wt% Pt	Pollutant(s)	*δ*	References
PD	0.4	Toluene	3	[102]
PD	0.2	EtOH	2.2	[64]
PD	0.2	Benzaldehyde	1.5	[103]
PD	0.2	Toluene	1.3	[103]
TR	1	Propene Propane	1.1 1.3	[89]
PD	0.5	Benzene	0.9	[104]
PD	0.5	Acetaldehyde	0.5 (25 °C) 3.8 (90 °C)	[105]
TR	0.1–2	TCE	0.125–0.07	[85]

*PD* photocatalytic deposition, *TR* thermal reduction with H_2_, *EtOH* ethanol, *TCE* trichloroethylene

As noted previously, there is no comprehensive study that shows both kinetic features, i.e. kfA and kFB, for a single pollutant, as illustrated in Fig. 14 by the bold line, which has been generated for such a system using the EPAO kinetic model. In the case of P25 TiO_2_, only the latter feature, kfB, appears to have been reported, using acid blue 9 or rhodamine B as the test pollutant [56] (see Fig. 9b and Table 3). An example of increasingly inhibition of PCO with increasing wt% Pt, see the broken black line profile illustrated in Fig. 14, is given in Table 2, by the work of Sun et al. [86], using phenol as the test pollutant. There appear to be no reported examples for P25 TiO_2_ in which the rate of PCO has been found to be invariant, and so *δ* = 1, at all wt% Pt values (see the broken red line in Fig. 14). However, there is at least one example in Table 2 where such ‘unaffected’ kinetics are likely to be exhibited, namely the photocatalytic oxidation of 2,4 dichlorophenoxy acetic acid, DCPAA, reported by Crittenden and his group [100], since even when the Pt loading was 1 wt%, these researchers found the PCO rate unchanged, and so *δ* = ca. 1 (see Table 3).

When surveying the values of *δ* reported for reaction (3) given in Table 3, it is not surprising that well-established SEDs, like methanol and ethanol, when used as the test pollutant, exhibit some of the highest values for *δ* listed, i.e. 7.8 and 4.2, respectively. Although, also in Table 3, it can be seen that a more modest value of *δ* of 2.4 was reported by the same workers for the same SEDs, under alkaline, pH 10.9, rather than neutral conditions [94]. This striking example of the effect of pH on *δ*, provides an appropriate reminder that there are many variables, some of which are listed in Table 2, which can affect PCO rate and so *δ*. As noted before, test organic pollutants that are hard to mineralise oxidatively might well be expected to exhibit no enhancement in activity with platinisation, as the RDS will not be reaction (4), the reduction of O_2_. Indeed, under such circumstances, the Pt NPs would be expected to promote electron–hole recombination, thereby inhibiting the PCO reaction and producing a *δ* versus wt% profile similar to that illustrated by the broken black line in Fig. 14. The sub-unity values for *δ* reported in Table 2 for the chlorinated solvents, chloroform and trichloroethylene (TCE) [94, 100], suggest that they are examples of such refractory organic test pollutants.

In contrast to the purification of water, reported examples PCO systems for air purification are less common, as indicated by the size of Table 4, compared with that of Table 3. The most surprising entry is that for toluene with its *δ* value of 3 [102], although others have reported it to be 1.3 [103], which appears a more likely value given its refractory nature [106]. The photocatalytic oxidation of acetaldehyde is interesting in that at room temperature, 25 °C, the system exhibits inhibition kinetics, with *δ* = 0.5, and yet at 90 °C, enhanced kinetics are observed, with *δ* = 3.8 [103]. Most of this ‘enhancement’ appears to be due to a loss of activity for reaction (13) by the TiO_2_, rather than a marked improvement in the rate exhibited by the P25 TiO_2_/Pt [103]. The authors of this work suggest that this loss is due to a poison that is formed in the dark on the surface of TiO_2_ and that it may be a polymeric species derived via a dark aldol condensation reaction [103]. This example, provides, once again, a timely reminder that the rate of reaction (13) depends on many different factors, making it difficult to compare and contrast results with confidence. The entry for TCE in Table 4 indicates the value of *δ* decreases with increasing wt% Pt and so is consistent with what was observed for refractory chlorinated solvents in aqueous solution and inhibition type kinetics.

## Conclusions

The PCO of organic pollutants in aqueous solution and in air by TiO_2_ is often enhanced by the deposition of 0.5–1.0 wt% of Pt NPs onto the surface of the photocatalyst; the enhancement typically lies in the range 0–8 and most often is 2–4. No comprehensive study of the rate of PCO as a function of wt% Pt showing enhancement and then subsequent fall with increasing wt% Pt has been reported in the literature to date, although the two kinetic features, namely the rise (kfA) and subsequent fall (kfB), have been captured in separate studies [56, 63] (see Fig. 9). These two kinetic features are very similar to those exhibited by the photocatalysed production of hydrogen by SEDs, like methanol, using TiO_2_/metal photocatalysts. The kinetic features of both systems are described very well by the EPAO kinetic model, which is based on an electric field that is set up between each Pt NP and the surrounding TiO_2_, which helps channel the photogenerated electrons to the Pt, which then mediates their subsequent reaction with O_2_. This model assumes the RDS for PCO is the reduction of O_2_ and so is not applicable to the PCO of refractory pollutants such as chlorinated solvents. Indeed, in the latter case, the Pt NPs appear to significantly enhance electron–hole recombination, so that in the presence of Pt, *δ* is always < 1 and decreases with increasing wt% Pt. This review on PCO does suggest that some noticeable enhancement (by a factor of 2–3) might be achieved using a very low (say, 0.01 wt%) loading of Pt and, therefore, this level of Pt might be worth incorporating into some commercial photocatalysts at no marked additional cost.

## Declarations

### Data availability

Data information can be obtained at 10.17035/d.2022.0162608211.
